# Suppression of *SDP1* Improves Soybean Seed Composition by Increasing Oil and Reducing Undigestible Oligosaccharides

**DOI:** 10.3389/fpls.2022.863254

**Published:** 2022-03-23

**Authors:** Jose A. Aznar-Moreno, Thiya Mukherjee, Stewart A. Morley, Dechassa Duressa, Shrikaar Kambhampati, Kevin L. Chu, Somnath Koley, Doug K. Allen, Timothy P. Durrett

**Affiliations:** ^1^Department of Biochemistry and Molecular Biophysics, Kansas State University, Manhattan, KS, United States; ^2^Donald Danforth Plant Science Center, St. Louis, MO, United States; ^3^United States Department of Agriculture, Agricultural Research Service, St. Louis, MO, United States

**Keywords:** lipase activity, oil, protein, raffinose family oligosaccharides, SDP1, soybeans

## Abstract

In developing soybean seeds, carbon is partitioned between oil, protein and carbohydrates. Here, we demonstrate that suppression of lipase-mediated turnover of triacylglycerols (TAG) during late seed development increases fatty acid content and decreases the presence of undigestible oligosaccharides. During late stages of embryo development, the fatty acid content of soybean seed decreases while the levels of the oligosaccharides raffinose and stachyose increase. Three soybean genes orthologous to the Arabidopsis lipase gene *SUGAR-DEPENDENT1* (*SDP1*) are upregulated at this time. Suppression of these genes resulted in higher oil levels, with lipid levels in the best lines exceeding 24% of seed weight. In addition, lipase-suppressed lines produced larger seeds compared to wild-type plants, resulting in increases of over 20% in total lipid per seed. Levels of raffinose and stachyose were lower in the transgenic lines, with average reductions of 15% in total raffinose family oligosaccharides observed. Despite the increase in oil, protein content was not negatively impacted and trended higher in the transgenic lines. These results are consistent with a role for SDP1 in turning over TAG to supply carbon for other needs, including the synthesis of oligosaccharides, and offer new strategies to further improve the composition of soybean seeds.

## Introduction

Soybeans are valued for their high protein content, approximately 40% of seed biomass, that provides the most adequate supply of amino acids for human and animal consumption compared to other plant protein sources (Wilson, 2004). In addition, soybeans produce a significant amount of oil, typically 20% of seed mass, supporting a market for soybean oil, the second most produced plant oil in the world (USDA, 2021). However, it has long been recognized that increasing the levels of either one of these valuable seed components comes at a cost to the other. Numerous studies on multiple soybean populations have demonstrated that soybean lines with high seed protein possess lower levels of oil, and seeds high in oil generally have lower protein (Johnson et al., 1955; Hymowitz et al., 1972; Wilcox, 1998; Wilcox and Shibles, 2001). Breaking this inverse correlation between oil and protein would improve soybean seed composition and help increase agricultural commodity production.

In addition to oil and protein storage reserves, soybeans also contain carbohydrates, which contribute less to the market value. Some carbohydrates, such as the oligosaccharides raffinose and stachyose, cannot be digested by monogastric animals, causing flatulence and reducing the energy available from the seed. These raffinose family oligosaccharides (RFOs) are therefore considered anti-nutritional compounds that detract from the value of the seed (Coon et al., 1990; Suarez et al., 1999; Valentine et al., 2017). RFOs are synthesized from sucrose by the sequential action of raffinose synthase and stachyose synthase. First, raffinose synthase transfers a galactosyl group from galactinol to sucrose to produce raffinose; stachyose is then formed in a similar manner from raffinose and galactinol by stachyose synthase. Consistent with this description, mutations in the soybean raffinose synthase gene *RS2* result in lower levels of raffinose and stachyose and higher levels of sucrose (Dierking and Bilyeu, 2008, 2009a). Likewise, a soybean line containing a short deletion in the stachyose synthase gene *STS* was associated with low levels of stachyose but increased amounts of raffinose (Qiu et al., 2015).

The final composition of a seed is determined by the supply of nutrients from the maternal plant and their subsequent modification through different metabolic pathways over the course of seed development (Allen and Young, 2013). Importantly, both the supply of sugars and amino acids from the maternal plant, as well as the enzymatic activity of different metabolic pathways, vary during seed development. For example, the amount of soybean seed exudate and the concentration of metabolites present is greatly reduced during late seed development (Kambhampati et al., 2021) and variations in gene expression and pathway enzyme levels over the course of seed development are well documented (Hajduch et al., 2005; Collakova et al., 2013; Li et al., 2015). In particular, in soybean, seed lipid levels decrease over late development whereas the levels of RFOs and insoluble seed components increase during the same time (Collakova et al., 2013; Kambhampati et al., 2020, 2021). Using stable isotope labeling to monitor metabolism, we recently demonstrated that gluconeogenesis was elevated during late soybean seed development and was synchronized with lipid turnover, possibly to provide carbon for carbohydrate synthesis (Kambhampati et al., 2021). We therefore hypothesized that preventing lipid turnover might simultaneously increase seed oil content while reducing the levels of undesirable RFO or other carbohydrates present in the seed. Importantly, the higher levels of oil would not come at the cost of reduced protein content because much of the carbon would be reallocated from carbohydrates to oil at this stage in development.

In germinating Arabidopsis seeds, the patatin-like lipase SUGAR-DEPENDENT1 (SDP1) and its close paralog SDP1-LIKE (SDP1L) hydrolyze stored TAG, releasing fatty acids as a source of energy and carbon skeletons for seedlings (Eastmond, 2006; Kelly et al., 2011). Based on expression levels and mutant phenotypes, SDP1 is responsible for the majority of TAG hydrolysis during germination, with SDP1L playing a more minor role (Kelly et al., 2011). SDP1 is expressed in different tissues, but transcript levels are highest during late seed development and in the resulting mature seeds (Kelly et al., 2011), suggesting that SDP1 might be responsible for TAG turnover during this same period of the life cycle. In support of this idea, the RNAi-mediated suppression of *SDP1* resulted in higher seed oil content in Arabidopsis (van Erp et al., 2014). Similarly, seed-specific suppression of *SDP1* orthologs in rapeseed, Jatropha and soybean also caused increased seed oil content in these species (Kelly et al., 2013; Kim et al., 2014; Kanai et al., 2019), though mechanistic alterations in carbon allocation have not been described.

Given the expression of *SDP1* late in Arabidopsis seed development and subsequent increases in oil content when this expression was reduced (Kelly et al., 2011; van Erp et al., 2014), we hypothesized that orthologs of SDP1 in soybean seed were responsible for TAG turnover and could provide carbon that enables RFO synthesis. To test this idea, we identified four *SDP1* homologs expressed in soybean seeds and used RNAi to suppress their expression. Similar to previous seed studies, we show that for multiple *SDP1*-suppressed lines, fatty acid content is increased. Importantly, levels of raffinose and stachyose are reduced, consistent with our hypothesis that the SDP1-mediated turnover of TAG provides carbon that enables less valued carbohydrate synthesis.

## Materials and Methods

### Plant Growth Conditions

Soybean (*Glycine max*) plants were grown in greenhouse with supplemental lighting to ensure a 14 h day/10 h night photoperiod, with day/night temperatures of 25°C/15°C. Pods were harvested at R5 (beginning seed), R6 (full seed), R7 (beginning maturity), R7.5 and R8 (full maturity) stages of reproductive development (Fehr and Caviness, 1977) from individual plants and placed directly on ice. The intermediate maturity stage of R7.5 was included because lipid breakdown and carbon reallocation are active at this point in development (Kambhampati et al., 2021). Seeds were removed from the pods, frozen in liquid nitrogen and stored at −80°C until analyzed. Transgenic soybeans (Williams82 background) were generated at the Plant Transformation Facility at Iowa State University using *Agrobacterium*-mediated transformation of seeds (Luth et al., 2015).

### Phylogenetic Analysis

Soybean SDP1 homologs were identified using BLASTP (Altschul et al., 1997) to search the soybean genome for sequences similar to Arabidopsis SDP1 (At5g04040) and SDP1L (At3g57140) protein sequences. An alignment of these amino acid sequences was performed using MEGA X program (Kumar et al., 2018). This alignment was used to generate a phylogenetic tree based on the neighbor-joining algorithm (Saitou and Nei, 1987), and the resulting phenogram was created using MEGA X.

### Gene Expression Analysis

Developing soybean seeds were harvested and the seed coat removed before grinding in liquid nitrogen using a precooled mortar and pestle. Total RNA was extracted using the RNeasy Plant Mini Kit (Qiagen) according to the manufacturer’s instructions and treated with DNaseI (Merck). cDNA was synthesized with the qScript cDNA SuperMix (Quanta Biosciences) from 1 μg total RNA. Quantitative PCR using specific primer pairs (Supplementary Table S3) and iTaq Universal SYBR Green SupermixI (Bio-Rad) was carried out in a CFX-96 real-time PCR system (Bio-Rad) to monitor the resulting fluorescence. Gene expression of lipase genes was normalized to the geometric mean of expression of the reference genes *ATP* (Glyma.12 g020500), *SKIP16* (Glyma.12 g051100) and *ELF1B* (Glyma.02 g276600 and Glyma.14 g039100; Hu et al., 2009).

### Double-Stranded RNAi Construction

RNAi hairpins driven by the soybean glycinin promoter and using the glycinin terminator were inserted between the EcoRI-HindIII sites in the plasmid pTF101.2 provided by the Plant Transformation Facility at Iowa State University. Full-length sequences for the glycinin promoter and terminator were amplified using the pKMS3glyprom-F/R and pKMS3gly-F/R primer pairs (Supplementary Table S4). The PCR products containing the restriction sites for the enzymes EcoRI and XmaI (promoter), or BamHI and HindIII (terminator), were cloned into the pTF101.2 vector (Supplementary Figure S5A). The PDK intron contained within the RNAi hairpin (Wesley et al., 2001) was amplified using the primers Pdk-F (containing XmaI and XbaI site) and Pdk-R (containing SacI and BamHI sites) and then inserted between the promoter and terminator by the XmaI and BamHI restriction sites (Supplementary Figure S5B). The two restriction sites generated for each side of the PDK intron were used to clone the amplified sense and antisense sequences for Glyma.02 g190000 and Glyma.19 g132900 obtained using primers in Supplementary Table S4 (Supplementary Figure S5C). The resulting vectors *sdp1-1* A and *sdp1-1* B that target Glyma.02 g190000, and *sdp1-3* C and *sdp1-3* D that target Glyma.19 g132900 were confirmed by sequencing prior to transformation into soybean plants.

### Seed Compositional Analysis

The content and composition of fatty acids, amino acids and oligosaccharides were determined for mature R8 seeds, harvested from two plants for each independent transgenic line. Six different pools of seeds were crushed and composition quantified for plant in a single collection where all lines, including wild-type controls, were grown at the same time. Seed fatty acid content was measured with the method described in Kambhampati et al. (2021). Briefly, fatty acid methyl esters (FAMEs) were synthesized from 10 mg of mature seeds using a standard acid-catalyzed method with 1 mg triheptadecanoin as an internal standard and then quantified by gas chromatography with flame ionization detection. Peptide hydrolysis and protein quantification was performed using an isotopic-dilution strategy for amino acid-based quantification (Kambhampati et al., 2019). Briefly, 4 mg of mature seeds were spiked with 20 pmol ^13^C ^15^N-labeled amino acid standard mix and hydrolyzed in 4 M methane sulfonic acid with 0.2% tryptamine. Following neutralization, amino acids were quantified with hydrophilic interaction chromatography coupled tandem mass spectrometry (HILIC-MS/MS) using a Shimadzu Prominence-xR high-performance liquid chromatography (HPLC) system connected to a SCIEX hybrid triple quadrupole-linear ion trap mass spectrometry with column and instrument settings described previously (Kambhampati et al., 2019). Absolute quantification of amino acids was determined based on calibration curves for each amino acid. Oligosaccharide extraction was performed as described previously (Dierking and Bilyeu, 2008). Sugars were extracted from 20 mg of seed in 50% ethanol but heating at 70°C; 10 μg ml^−1^ ribotol was added as an internal standard. Sugars were quantified using a Shimadzu Prominence-xR high-performance liquid chromatography (HPLC) system connected to a SCIEX hybrid triple quadrupole-linear ion trap mass spectrometry with column and instrument settings described previously (Kambhampati et al., 2021). Statistical differences between genotypes were determined using Student’s *t*-test with Welch’s correction for unequal variances on GraphPad Prism.

### Germination Assay

Twenty-four seeds per genotype with 12 seeds per plant were planted in potting soil at a depth of 1 in. After planting, water was sprayed to settle soil over the seeds and the trays were then placed in greenhouse with day/night conditions of 14/10 h and 25/23°C. Seedling germination was recorded at the same time each day and was defined by the emergence of the hypocotyl at the surface of the soil. Kaplan–Meier curves of seeds from wild-type and transgenic plants were estimated using the survival package in R. Right censoring was incorporated if the seed had not yet germinated at the end of the measured timeframe. A Cox proportional hazard model was fitted to estimate hazard coefficient of each genotype (Cox, 1972). A plant effect term was tested and shown to not have a significant effect (*p* > 0.05) and therefore was not included as an effect in subsequent analyses. Hazard ratio tests for all transgenic lines were performed against wild-type plants. Using the germination hazard coefficients, Pearson correlation was measured against *SPD1* normalized relative expression.

## Results

### The Soybean Genome Possesses Four *SDP1* Orthologs Expressed During Late Seed Development

**Figure 1:**
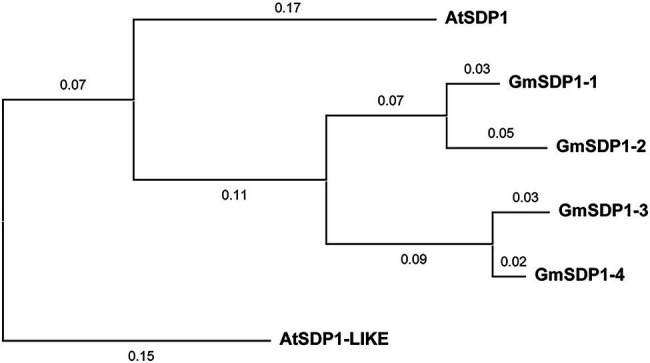
Phylogenetic analysis of soybean proteins similar to Arabidopsis SDP1. The phylogenetic tree was constructed with MEGA X using the neighbor-joining method. Numbers on branches represent evolutionary distance in substitutions per amino acid. AtSDP1-LIKE (At3g57140) is the Arabidopsis paralog most similar to AtSDP1 (At5g04040) and was included for comparison. GmSDP1-1, Glyma.02 g190000; GmSDP1-2, Glyma.10 g105200; GmSDP1-3, Glyma.19 g132900; GmSDP1-4, Glyma.03 g130900.

The suppression of SDP1 orthologs in the seeds of different plant species increases their fatty acid content (Kelly et al., 2013; Kim et al., 2014; Kanai et al., 2019), implying that these lipases might mediate the redistribution of carbon from TAG during late soybean seed development. We identified four genes encoding lipases with high homology to Arabidopsis SDP1 present in the soybean genome. Phylogenetic analysis indicated that all four soybean SDP1 homologs are more closely related to AtSDP1 than AtSDP1-LIKE (Figure 1). Amino acid alignments revealed that similar to AtSDP1 and AtSDP1-like, all four GmSDP1 proteins possess a predicted patatin-like domain containing a conserved GXSXG serine esterase motif (Supplementary Figure S1).

**Figure 2:**
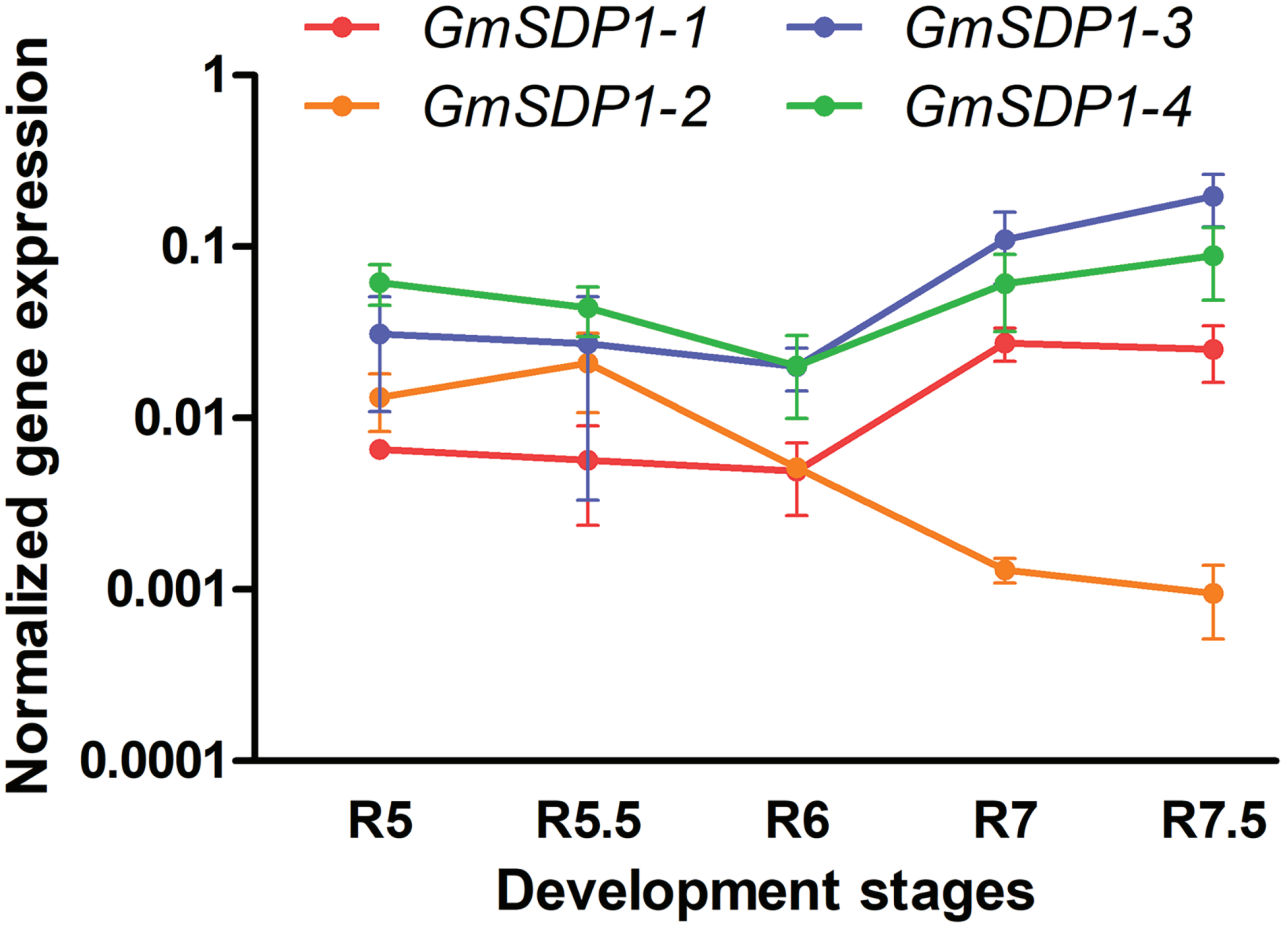
Expression of soybean *SDP1* genes during seed development. *GmSDP1* transcript levels were quantified using RT-qPCR with paralog-specific primers and normalized to the geometric mean of the expression of soybean *ATP* and *SKIP16* genes. Values represent the mean ± SD of normalized expression levels of seeds collected from three individual plants.

We quantified the expression of the four *GmSDP1* genes in wild-type Williams 82 seed from R5 (beginning seed) to R7.5 (maturation) stages using RT-qPCR. The fatty acid accumulation in these samples was previously shown to decrease from a maximum of 40 mg/seed at R7 to 30 mg/seed when mature (Kambhampati et al., 2021). In addition, stachyose and raffinose levels in these seeds increased during late seed development. Quantification of transcript levels revealed that all four *SDP1* homologs were strongly expressed in soybean seeds, with transcript levels for three genes (*GmSDP1-1*, *GmSDP1-4,* and *GmSDP1-3*) increasing during late seed development (Figure 2). In contrast, the expression of *GmSDP1-2* decreased in the late stages. Thus, at the latest seed stage analyzed (R7.5), *GmSDP1-3* and *GmSDP1-4* possessed the highest expression with *GmSDP1-2* having the lowest expression. Further, the highest expression of *GmSDP1-1*, *GmSDP1-4,* and *GmSDP1-3* coincided with the decrease in fatty acid accumulation observed during late seed development (Kambhampati et al., 2021), implicating a role for these three genes in TAG and eventual fatty acid turnover.

### Suppression of *GmSDP1* Orthologs Increases Seed Fatty Acid Content

**Figure 3:**
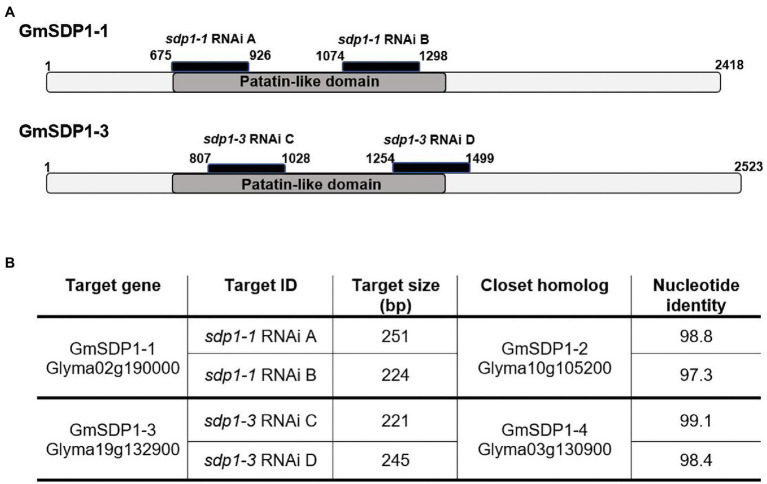
RNAi target design for SDP1 lipases in soybean. **(A)** Schematic representation of the two lipase genes selected for suppression, showing the region of the RNAi target sequences in black and patatin-like domains in dark grey. Numbers indicate the position of the sequence from the start codon. **(B)** Table showing identity values between the target sequences and corresponding regions of the closest *GmSDP1* paralog.

Based on their expression patterns during seed development (Figure 2) and their phylogenetic relationship (Figure 1), we designed RNAi hairpins to target the expression of all four soybean *SDP1* homologs (Figure 3A). Due to the high homology between pairs of genes (Figure 1), the RNAi hairpin designed for *GmSDP1-1* displayed high identity (more than 98%) with *GmSDP1-2* allowing the simultaneous targeting of both genes (Figure 3B; Supplementary Table S1). Likewise, as *GmSDP1-3* possessed high sequence identity (>99%) with *GmSDP1-4*, both genes were targeted together. To increase the chances of effective suppression, we designed two different RNAi hairpins for each pair of *GmSDP1* genes (Figure 3). Each hairpin also possessed considerable overlapping identity (typically >90%) with the other pair of *GmSDP1* paralogs (Supplementary Table S1). The hairpins were expressed under control of the strong seed-specific glycinin promoter to minimize pleiotropic effects at other developmental stages, particularly given the role of SDP1 in mobilizing TAG reserves during germination (Eastmond, 2006; Kelly et al., 2011). The four different *GmSDP1* suppression constructs were transformed into soybean (Williams 82 background).

**Figure 4:**
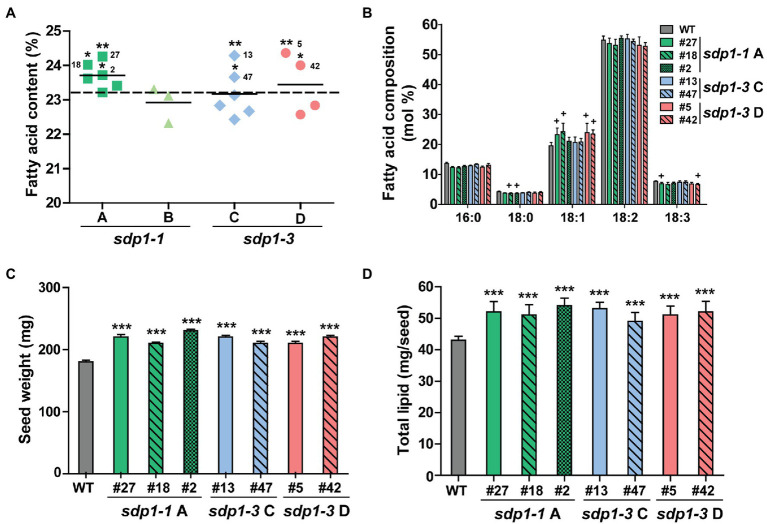
Transgenic lines targeting *GmSDP1* expression possess seeds with increased high fatty acid content. **(A)** Scatter plot showing the fatty acid content of seeds containing different constructs targeting *SDP1* expression. Each data point corresponds to an independent transgenic line and represents the mean fatty acid content as a % of dry seed weight of T3 seeds from two plants for each line. Solid horizontal lines represent the average fatty acid content for each construct. The horizontal dashed line indicates the mean fatty acid content of seeds harvested from two control wild-type plants. Numbers denote the specific lines chosen for further compositional analysis. Statistically significant differences between wild-type and transgenic lines were determined using Student’s *t*-test; **, *p* < 0.05; *, *p* < 0.3. **(B)** Mean fatty acid composition of T3 seed harvested from two plants each for select transgenic lines. Error bars represent SD. Crosses indicate significant differences with wild-type seed (+, *p* ≤ 0.001; Student’s *t*-test). **(C)** Average seed weight. Values represent the mean ± SD of 45 seeds collected from two plants for each line. ***, *p* < 0.001 based on a two-tailed t-test. **(D)** Seed fatty acid content calculated on a per seed basis. Values represented the mean ± SD. ***, *p* < 0.001 based on a two-tailed *t*-test.

After identifying lines displaying homozygous expression of each of the four constructs, we quantified the total fatty acid content and composition in mature T3 seeds (Figures 4A,B; Supplementary Table S2). With the exception of one line with wild-type fatty acid levels, seed expressing *sdp1-1 RNAi A* targeting *GmSDP1-1* and *GmSDP1-2* all possessed higher fatty acid levels compared to wild-type controls. Here, on average, seed from the best transgenic line had an oil content of 24.3% of seed mass, while wild-type seed had an oil content of 23.3% (Figure 4A). However, the targeting of the same pair of genes by *sdp1-1 RNAi B* did not result in notable increases, with two lines producing seed with fatty acid levels similar to that of wild type, and one line possessing lower seed fatty acid content. The targeting of *GmSDP1-3* and *GmSDP1-4* by *sdp1-3 RNAi C* and *sdp1-3 RNAi D* did result in some lines that produced higher levels of fatty acids, up to 24.4% of seed mass in the highest lines, but other lines possessed lower levels. The successful RNA interference of some constructs became obvious as one design (*sdp1-1 A*) consistently increased oil whereas the effect of other constructs was less uniform. The fatty acid composition of seed from most of the transgenic lines was unchanged relative to wild-type seeds (Figure 4B; Supplementary Table S2). In some of the lines producing seed with the highest fatty acid content, we did notice small but significant increases in 18:1 with concomitant reductions in 18:2 and 18:3. Further detailed analysis of seeds with high fatty acid content revealed an increase in seed weight, with transgenic seeds ranging from 208 to 226 mg/seed compared with wild-type seeds with an average mass of 183 mg/seed (Figure 4C). Similar to our results, the suppression of *GmSDP1* in a different soybean background also resulted in increased seed fatty acid content, higher 18:1 levels and increased seed weight (Kanai et al., 2019). As we have noted previously (Kambhampati et al., 2021), comparing metabolite amounts on a per seed basis more accurately describes changes in accumulation over seed development. Thus, the increase in average seed weight, combined with the higher fatty acid content of these lines, resulted in higher levels of fatty acid per seed, with increases of over 20% compared to wild-type observed in some lines (Figure 4D).

**Figure 5:**
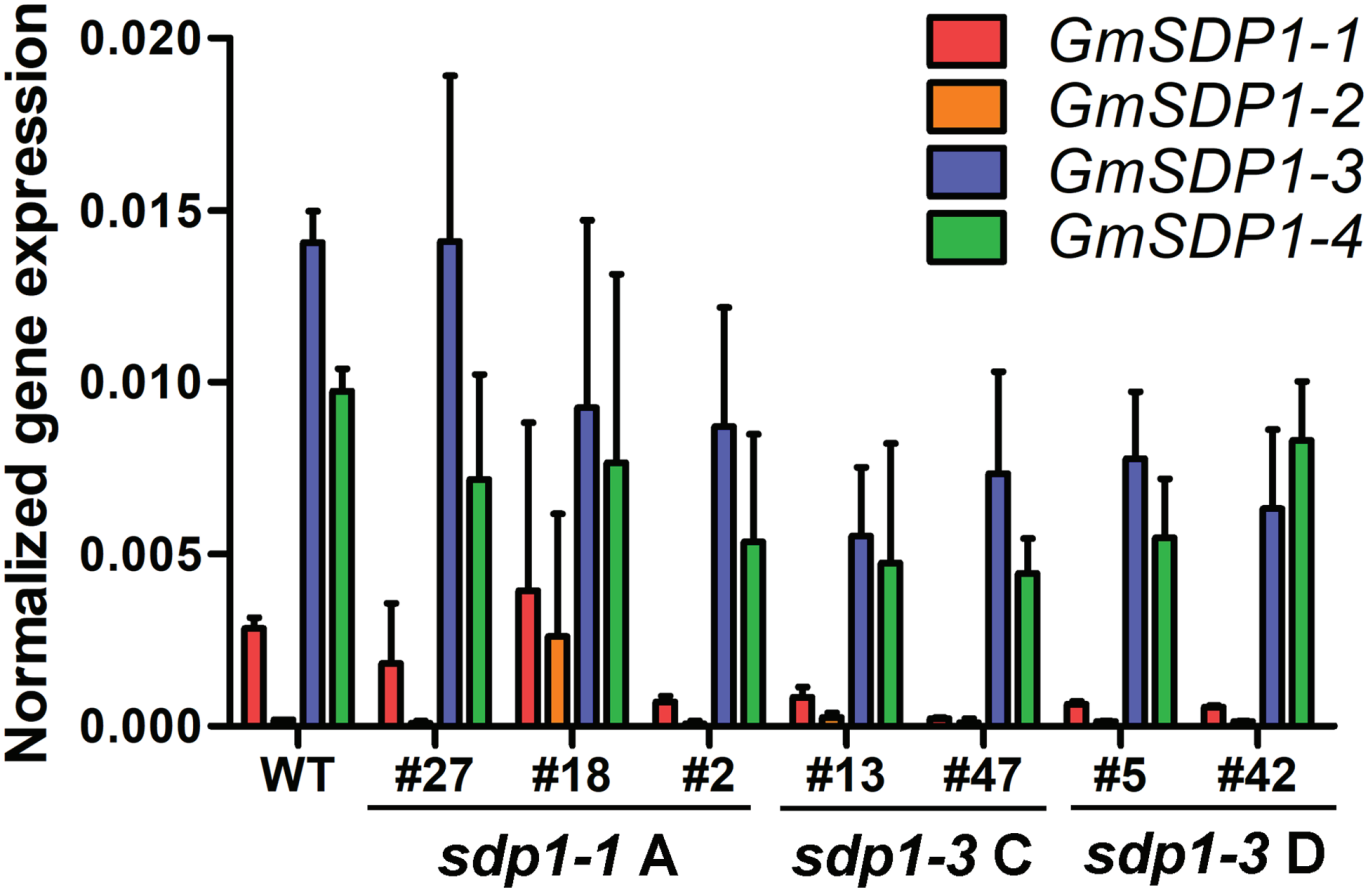
*GmSDP1* expression is suppressed in transgenic lines. *GmSDP1* expression in R7 seeds was quantified using RT-qPCR with paralog-specific primers and normalized to the geometric mean of the reference genes *ELF1B* and *SKIP16*. Values represent the mean expression values for seeds collected from two individual plants for each line.

To confirm that these increases in fatty acid content were being caused by the RNAi-mediated suppression, we quantified transcript levels of the four *GmSDP1* genes in developing T3 seeds collected from lines possessing the highest oil content (Figure 4A). *GmSDP1* gene expression was measured in R7 seeds because this stage of development possessed the highest expression in wild-type seeds (Figure 1). In most lines, transcript levels of the two highest expressed paralogs, *GmSDP1-3* and *GmSDP1-4*, were reduced by 20–75% (Figure 5). The one exception was *sdp1-1A #27* where *GmSDP1-3* did not appear to be suppressed. Similarly, expression of *GmSDP1-1* was lower in all lines compared to wild type, with the exception of *sdp1-1A #18*. *GmSDP1-2* transcript levels were also generally lower in transgenic seed; however, given that this gene is expressed at much lower levels in wild-type seeds compared to its paralogs (Figure 2B), accurate quantification of even lower levels was difficult. While the RNAi hairpins were designed to target pairs of *GmSDP1* paralogs (Figure 3), typically all four *GmSDP1* genes were suppressed in most transgenic lines (Figure 5). This result is not too surprising given that each hairpin possesses sufficient homology to potentially silence all four *GmSDP1* paralogs (Supplementary Table S1).

### Suppression of *GmSDP1* Orthologs Results in Reduced Seed Raffinose and Stachyose Content

**Figure 6:**
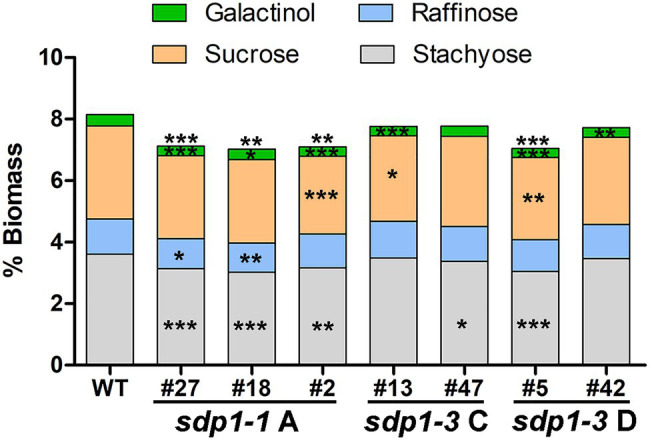
Suppression of *GmSDP1* reduces seed carbohydrate content. Mean carbohydrate content as a % of dry seed weight of T3 seed harvested from two plants each for select transgenic lines. Asterisks indicate significant differences compared to wild-type seed (*, *p* ≤ 0.05; **, *p* ≤ 0.01; ***, *p* ≤ 0.001; Student’s *t*-test).

We selected transgenic lines producing seed with the highest fatty acid content for further compositional analysis. In these lines, total levels of soluble carbohydrates were significantly lower in the seeds from most of the *GmSDP1*-suppressed lines (Figure 6). Much of this reduction in carbohydrates was caused by significantly lower levels of stachyose in the transgenic seeds. Raffinose content was also reduced in most of the lines but these differences were only significant for two lines, both of which expressed *sdp1-1 RNAi A*. Importantly, these two lines with significantly lower seed raffinose content also possessed lower stachyose content, such that total RFO content in the seed was reduced on average by 15% compared to wild-type seed levels. We also quantified the levels of sucrose and galactinol, the precursors of raffinose and stachyose. Compared to the wild-type controls, the amount of galactinol was significantly reduced in the seeds of all but one transgenic line. Sucrose levels tended to be lower in the seeds of the transgenic lines when compared to wild type, but these reductions were only significant in three lines. Seed protein content trended higher in these lines compared to wild type, but the increases were not statistically significant (Supplementary Figure S2A). There were only minor differences in amino acid composition (Supplementary Figure S2B).

### Transgenic Seeds Germinated Slower Than Wild-Type Seeds

**Figure 7:**
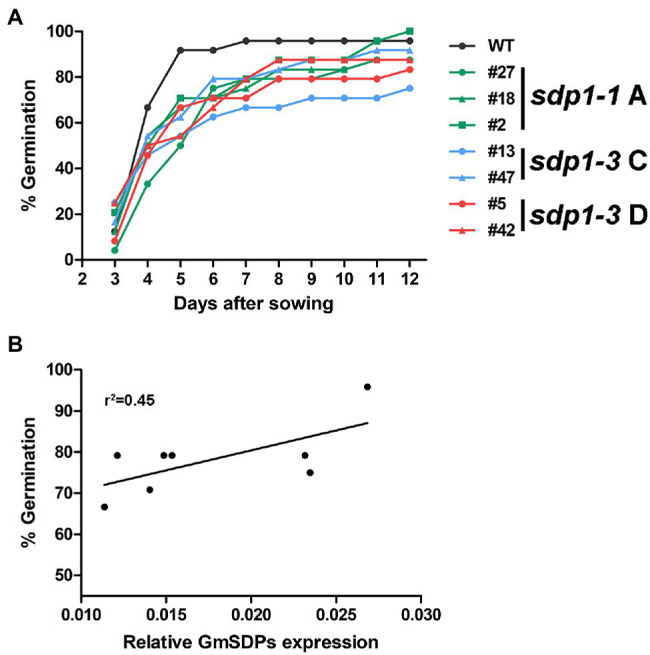
Suppression of *GmSDP1* delays germination. **(A)** Germination rates of seeds from wild-type (WT) and *GmSDP1*-suppressed transgenic plants. Values shown represent the mean germination rate of seeds harvested from two plants for each genotype. **(B)** Correlation between germination rate and total *GmSDP1* expression in R7 seeds.

Given the role of SDP1 in mobilizing TAG storage reserves in germinating Arabidopsis seeds (Eastmond, 2006; Kelly et al., 2011) we quantified germination efficiency in wild-type and *GmSDP1*-suppressed seeds. Seeds from the transgenic plants germinated slower than wild-type seeds (Figure 7A). For example, 5 days after sowing, only 50–70% of the transgenic seed had emerged from the soil whereas ~90% of the wild-type seed had emerged. However, by 12 days after sowing, seeds from most of the transgenic lines had achieved germination rates between 90 and 100%, similar to that of wild-type seed (~95% germination). Based on a Cox proportional hazard model, the difference in germination efficiency between *sdp 1–1A # 27*, *sdp 1–3 #13* and wild type was statistically significant (Supplementary Figure S3). Interestingly, the observed delay in germination was correlated with overall *GmSDP1* expression, with the lines possessing the lowest levels of seed *GmSDP1* expression at R7 also having the lowest germination rates (Figure 7B). Once germinated though, the transgenic lines possessed no obvious visual phenotypes compared to wild-type control plants (Supplementary Figure S4).

## Discussion

### Tag Turnover Mediated *via* SDP1 Provides Carbon for RFO Synthesis

Our earlier work suggested that the hydrolysis of TAG during late seed development supported the synthesis of insoluble carbohydrates, including undesirable RFOs (Kambhampati et al., 2021). Based on their expression in late seed development (Figure 2C), we hypothesized that lipases encoded by the *GmSDP1* family of genes (Figure 1) were likely to play a key role in rerouting carbon from TAG to RFOs. Further, the seed-specific suppression of *SDP1* orthologs in multiple species, including soybean, has been shown to increase seed oil content (Kelly et al., 2013; Kim et al., 2014; Kanai et al., 2019), consistent with a role for SDP1 in turning over TAG to provide precursors for other seed components. However, in these previous studies, the effects on other components of the seed were not rigorously examined. Suppression of *SDP1* expression in rape and jatropha caused a small decrease in seed protein content (Kelly et al., 2013; Kim et al., 2014), but effects on carbohydrate content and composition were not investigated. Similar to these previous studies, we show that suppression of *GmSDP1* paralogs during seed development results in increased fatty acid content, particularly when measured on a per seed basis (Figure 4). Importantly, we demonstrate that in these seeds with increased fatty acid content, the levels of stachyose and raffinose are lower, with reductions in stachyose being more significant (Figure 6). Further, amounts of the RFO precursors galactinol and sucrose were also affected in the transgenic seeds. Galactinol levels in particular were significantly lower in all but one of the lines.

**Figure 8:**
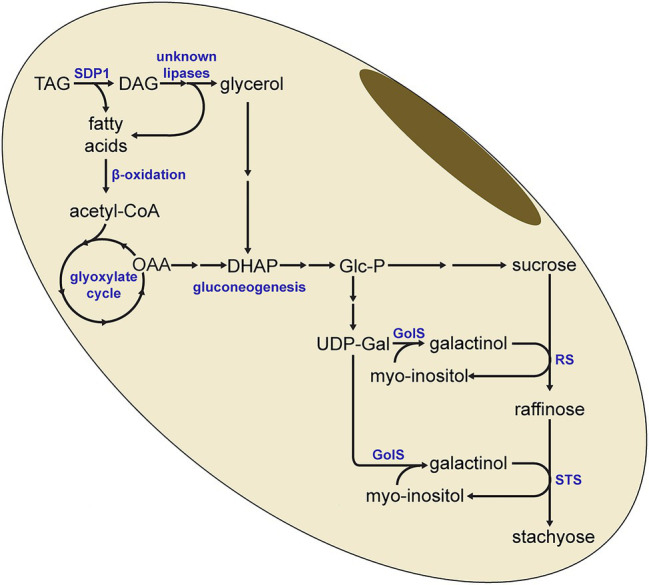
Model for the redistribution of carbon from triacylglycerol to raffinose and stachyose. SDP1-mediated hydrolysis of triacylglycerol (TAG) yields fatty acids, which can be converted to glucose-phosphate (Glc-P) through β-oxidation, the glyoxylate cycle and gluconeogenesis. Glc-P is then synthesized to UDP-Galactose (UDP-Gal) and sucrose, the precursors for raffinose and stachyose. It is possible for diacylglycerol (DAG) to be further hydrolyzed to provide additional fatty acids, as well as glycerol, for conversion to glucose. In this simplified pathway, not all enzymatic steps or intermediate metabolites are indicated. DHAP, dihydroxyacetone phosphate; GolS, Galactinol Synthase; OAA, oxaloacetate; RS, raffinose synthase; STS, stachyose synthase.

The increased lipid levels and the lower RFO levels are therefore consistent with a model where the SDP1-mediated breakdown of TAG results in the supply of carbon for RFO synthesis (Figure 8). Here, fatty acids derived from TAG hydrolysis would eventually generate carbohydrates through the combined action of β-oxidation, the glyoxylate cycle and gluconeogenesis. Our previous work has shown that gluconeogenesis is increased during late seed development and is synchronized with lipid turnover (Kambhampati et al., 2021), in line with this model. It is unclear if the diacylglycerol (DAG) resulting from the SDP1-mediated hydrolysis of TAG is further broken down to monoacylglycerol (MAG) or even to the glycerol backbone, which could feed into gluconeogenesis *via* dihydroxyacetone phosphate (DHAP; Figure 8). If this is the case and TAG is completely hydrolyzed to fatty acids and glycerol, then other lipases will also need to be involved in this pathway as SDP1 likely does not hydrolyze DAG and monoacylglycerol (MAG). Arabidopsis SDP1 possesses much lower activity *in vitro* with DAG and is inactive with MAG (Eastmond, 2006). In addition, Arabidopsis *sdp1 sdp1L* double mutants do not possess defects in DAG- or MAG-lipase activity (Kelly et al., 2011), suggesting that other genes encode these activities. While the DAG- and MAG-lipases responsible for the complete breakdown of stored TAG in adipocytes have been identified in mammals (Fredrikson et al., 1981, 1986; Eichmann et al., 2012), orthologous enzymes in plants remain less well characterized. Here, an Arabidopsis MAG-lipase that is expressed in germinating seeds and that localizes to the surface of oil droplets is likely the best candidate to play a role in storage lipid breakdown (Kim et al., 2016).

### Suppressing *SDP1* Affects RFO Synthesis

In the transgenic seeds, the levels of stachyose tended to be more affected than those of raffinose (Figure 6), consistent with the order of the RFO synthesis pathway where raffinose and then stachyose are formed from sucrose through the sequential addition of galactinol (Figure 8). Based on our results, it is tempting to speculate that breakdown of TAG might be particularly important for the provision of galactinol for the synthesis of raffinose and stachyose. As the synthesis of stachyose occurs downstream of raffinose, reductions in the supply of galactinol are more likely to affect levels of stachyose than raffinose, consistent with our observations (Figure 6). Other work has also shown that the supply of galactinol is important for RFO synthesis, particularly stachyose. For example, in soybean lines containing mutations in genes encoding galactinol synthase, the stachyose content decreased whereas raffinose content increased (Le et al., 2020). Similar to our observations, these results imply a distinction in the supply of galactinol for the synthesis of stachyose versus that of raffinose. Future work is therefore needed to elucidate the supply of precursors to different galactinol synthase isoforms, possibly spatially distinct sources, and whether these might be derived from the breakdown of TAG.

Currently, we cannot exclude the possibility that SDP1-mediated TAG breakdown also provides carbon for sucrose synthesis. Here it is relevant to note that sucrose levels are significantly lower in some transgenic lines. Consistent decreases in sucrose may not be apparent because reductions in raffinose and stachyose synthesis caused by limitations in galactinol availability could actually increase levels of the sucrose precursor as the pathway gets backed up. Indeed, increases in sucrose have been observed in mutant lines containing mutations in the raffinose synthase gene *RS2* (Dierking and Bilyeu, 2008, 2009a).

Though prior work is supportive of flux from TAG to hexose-derived products (Kambhampati et al., 2021), the reduction in oligosaccharide content when SDP1-mediated TAG turnover is suppressed, does not fully balance the gain in lipid. The changes in lipid level that are approximately 1% of biomass cannot be completely ascribed to the summed changes in oligosaccharides and sucrose which result in slightly less (i.e., ~0.9% biomass) because the composition of carbohydrates and lipids is different. Soybean oil is comprised of 77% carbon and a typical glucose polymer is approximately 43% carbon depending on the relative amounts of oligosaccharides and sucrose. This suggests that in wild-type seeds, some carbon from TAG turnover might be respired for energy production or that other insoluble carbohydrates are also impacted (Kambhampati et al., 2021). Finally, it is important to note that the seed composition of the *GmSDP1*-suppressed lines (Figures 4, 6) was based on analysis of mature seeds and therefore reflects end-point measurements of metabolite levels resulting from metabolism throughout seed development. Thus, future work to elucidate the allocation of carbon from TAG to other aspects of central and storage reserve metabolism will need to investigate seed composition and carbon flux at different stages of seed development.

### *SDP1* Expression Is Required for Rapid Seed Germination

Seeds from the transgenic lines germinated slower compared to wild type, but overall germination efficiency was similar (Figure 7). While RFO accumulation has been associated with the acquisition of desiccation tolerance in maturing seeds and might play a role during early germination (Blackig et al., 1996; Obendorf, 1997; Blöchl et al., 2007), the observed differences in germination are most likely caused by the suppression of *SDP1* rather than reductions in RFOs. First, similar to our observations, seeds from Arabidopsis *sdp1 sdp1l* double mutants also germinate slower but eventually attain a comparable germination rate to wild-type seeds (Kelly et al., 2011). Second, a soybean mutant line with low RFO levels was not affected in germination compared to wild-type controls (Dierking and Bilyeu, 2009b).

The effect on germination is somewhat surprising given that the RNAi hairpins designed to suppress *SDP1* were driven by the seed-specific glycinin promoter to minimize effects at other stages of plant development. However, similar results have been noted previously. For example, suppression of *SDP1* in developing rapeseed resulted in reduced fatty acid hydrolysis during germination (Kelly et al., 2013). One explanation for these results is that SDP1 is required at the start of germination to ensure rapid TAG mobilization. When expression is suppressed during seed development, the resulting seeds incur a delay while synthesizing sufficient SDP1 lipase activity. Alternatively, it is possible that even though it is considered a seed-specific promoter, the glycinin promoter might be expressed at low levels in other tissues, including germinating seeds, similar to what has been noted for other “seed-specific” promoters (Zakharov et al., 2004; Furtado et al., 2008).

## Conclusion

In summary, we demonstrate that targeting the expression of SDP1 lipases during soybean seed development simultaneously increases seed fatty acid content while reducing RFO content. Protein content was slightly increased as a consequence of available carbon late in development not used for carbohydrate biosynthesis. These results support the idea that the SDP1-mediated turnover of seed oil during late seed development may contribute to less valuable storage reserves and suggest ways of further improving soybean seed composition through targeting specific metabolic steps during seed development.

## Data Availability Statement

The raw data supporting the conclusions of this article will be made available by the authors, without undue reservation.

## Author Contributions

JA-M, SK, DA, and TD conceived and designed the study. TM, JA-M, SM, DD, KC, and SK designed and performed the experiments and analyzed the data. JA-M, TM, DA, and TD wrote the paper. All authors edited and approved the final manuscript.

## Funding

This work was supported by the United Soybean Board (Projects #1820-152-0134, #2020-152-0103, and #2220-152-0103), the USDA Agriculture Research Service, by the USDA National Institute of Food and Agriculture, Hatch/Multi-State project 1013013 and grant 2017-67013-26156, National Institutes of Health award U01 CA235508, and National Science Foundation award IOS-1829365. Support for the acquisition of the 6500 QTRAP LC–MS/MS was also provided by the National Science Foundation (NSF-DBI #1427621). This is contribution number 22-091-J from the Kansas Agricultural Experiment Station.

## Conflict of Interest

The authors declare that the research was conducted in the absence of any commercial or financial relationships that could be construed as a potential conflict of interest.

## Publisher’s Note

All claims expressed in this article are solely those of the authors and do not necessarily represent those of their affiliated organizations, or those of the publisher, the editors and the reviewers. Any product that may be evaluated in this article, or claim that may be made by its manufacturer, is not guaranteed or endorsed by the publisher.
