# Supplementary material for: Suppression of SDP1 Improves Soybean Seed Composition by Increasing Oil and Reducing Undigestible Oligosaccharides
Source: Front Plant Sci. 2022 Mar 23;13:863254. doi: 10.3389/fpls.2022.863254 (PMC8983916; doi:10.3389/fpls.2022.863254)
Supplement: Supplementary file 1 [file Data_Sheet_1.PDF]

Supplementary Material

```
             ....|....| ....|....| ....|....| ....|....| ....|....| ....|....| ....|....| ....|....| ....|....| ....|....| ....|....|
                   225        235        245        255        265        275        285        295        305        315        325
AtSDP1       LSFMHETRHA FGRTALLLSG GASLGAFHVG VVRTLVEHKL LPRIIAGSSV GSIICAVVAS RSWPELQSFF ENSLHSLQFF DQLGGVFSIV KRVMTQGALH DIRQLQCMLR
AtSDP1-like  LSFMHETRHA YGRTALLLSG GASLGAFHLG VVKTLVEHKL LPRIIAGSSV GSVMCAVVGT RSWPELQSFF EGSWHALQFF DQMGGIFTTV KRVMTQGAVH EIRHLQWKLR
GmSDP1-1     LSFMHETRHA FGRTALLLSG GASLGAFHVG VVKTLVEHKL LPRIIAGSSV GSIMCSIVAT RSWPELQSFF EDSLHSLQFF DQMGGIFTVV KRVTTYGAVH EIRQLQMLLR
GmSDP1-2     LSFMHETRHA FGRTALLLSG GASLGAFHVG VVKTLVEHKL LPRIIAGSSV GSIMCSIVAT RSWPELQSFF EDSLHSLQFF DQMGGIFTVV KRVTTYGAVH EIRQLQMLLR
GmSDP1-3     LAFMHETRHA FGRTALLLSG GASLGASHVG VVKTMVEHKL MPRIIAGSSV GSIMCAVVAT RTWPELQSFF EDSWHSLQFF DQMGGIFAVV KRVTTLGAVH EIRQLQMMLR
GmSDP1-4     LAFMHETRHA FGRTALLLSG GASLGASHVG VVKTLVEHKL MPRIIAGSSV GSIMCAVVAT RTWPELQSFF EDSWHSLQFF DQMGGIFAVV KRVTTLGAVH EIRQLQMMLR
             *:******** :********* ****** *:* **:*:***** :********* **::*::*.: *:******** *.* *:**** **:**:*: * *** * **:* :**:**  **

             ....|....| ....|....| ....|....| ....|....| ....|....| ....|....| ....|....| ....|....| ....|....| ....|....| ....|....|
                   335        345        355        365        375        385        395        405        415        425        435
AtSDP1       NLTSNLTFQE AYDMTGRILG ITVCSPRKHE PPRCLNYLTS PHVVIWSAVT ASCAFPGLFE AQELMAKDRS GEIVPYHPPF NLDPEVGTKS SSGRRWRDGS LEVDLPMMQL
AtSDP1-like  NLTNNLTFQE AYDITGRILG ITVCSLRKHE PPRCLNYLTS PHVVIWSAVT ASCAFPGLFE AQELMAKDRT GEIVPYHPPF NLDPEEG--S ASVRRWRDGS LEMDLPMIQL
GmSDP1-1     HLTSNLTFQE AYDMTGRILG ITVCSPRKHE PPRCLNYLTS PHVVIWSAVT ASCAFPGLFE AQELMAKDRS GEIVPYHPPF NLGPEKG--S TSVRRWRDGS LENDLPMMQL
GmSDP1-2     NLTSNLTFQE AYDMTGRILG ITVCSPRKHE PPRCLNYLTS PHVVIWSAVT ASCAFPGLFE AQELMAKDRS GEIVPYHPPF NLGPEKG--S TSVRRWRDGS LEMDLPMMQL
GmSDP1-3     HLTSNLTFQE AYDMTGRILG ITVCSPRKHE PPRCLNYLTS PHVVIWSAVT ASCAFPGLFE AQELMAKDRS GEIIPYHPPF NLGPEEG--S TPARRWRDGS LEIDLPMMQL
GmSDP1-4     HLTSNLTFQE AYDMTGRILG ITVCSPRKHE PPRCLNYLTS PHVVIWSAVT ASCAFPGLFE AQELMAKDRS GEIVPYHPPF NLGPEEG--S TPVRRWRDGS LEIDLPMMQL
             :**.****** ***:****** ***** **** ********** ********** ********** *********: ***:****** **.** *  * :. ******* ** ****:**

             ....|....| ....|....| ....|....| ....|....| ....|....| ....|....| ....|....| ....|....| ....|....| ....|....| ....|....|
                   445        455        465        475        485        495        505        515        525        535        545
AtSDP1       KELFNVNHFI VSQANPHIAP LLRLKDLVRA YGGRFAAKLA HLVEMEVKHR CNQVLELGFP LGGLAKLFAQ EWEGDVTVVM PATLAQYSKI IQNPTHVELQ KAANQGRRCT
AtSDP1-like  KELFNVNHFI VSQANPHIAP FLRMKEFVRA CGGRFAAKLA QLAEMEVKHR CNQVLELGLP LREVASLFAQ EWEGDVTIVM PATFSQYLKI IQNPSNVEIQ KAANQGRRCT
GmSDP1-1     KELFNVNHFI VSQANPHIAP LLRLKEFVRA YGGNFAAKLA HLAEMEVKHR CNQVLELGFP LGGLAKLFAQ DWEGDVTVVM PATLAQYLKI IQNPSYVELQ KAANQGRRCT
GmSDP1-2     KELFNVNHFI VSQANPHIAP LLRFKEIIRA YGGNFAAKLA HLAEMEVKHR CNQVLELGFP LGGLAKLFAQ DWEGDVTVVM PATPAQYLKI IQNPSYVELQ KAANQGRRCT
GmSDP1-3     KELFNVNHFI VSQANPHIAP LLRLKEFVRT YGGNFAAKLA HLVEMEVKHR CNQILELGFP LGGLAKLFAQ DWEGDVTVVI PATLAQYTKI IQNPSYGELQ KATNQGRRCT
GmSDP1-4     KELFNVNHFI VSQANPHIAP LLRLKEFVRT YGGNFAAKLA HLVEMEVKHR CHQILELGFP LGGLAKLFAQ DWEGDVTVVI PATLAQYTKI IQNPSYGELQ KAANQGRRCT
             ********** ********** :**:*:::*:  **.****** :*.******* *:*:****:* *  :*.**** :******:*: *** :** ** ****:   *:* **:*******
```

Figure S1. GmSDP1 paralogs possess high amino acid sequence identity with Arabidopsis SDP1. The patatin-like domain is underlined and the conserved GXSXG serine esterase motif is outlined with a box. * indicates amino acid identity for all six sequences.

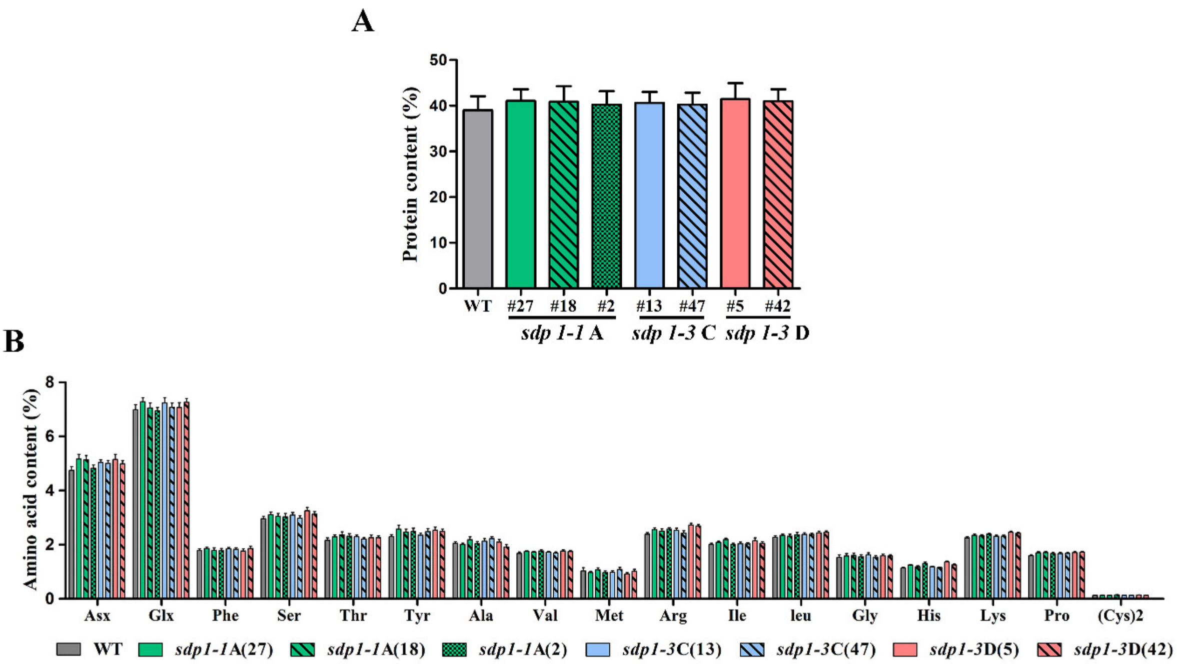

Figure S2. Protein content of mature seed of *GmSDP1*-suppressed transgenic lines. (**A**) Total protein content as a percentage of seed weight was quantified for select transgenic lines and wild-type (WT) plants. (**B**) Amino acid composition of wild-type and *GmSDP1*-suppressed transgenic lines. Values represent the mean ± SD of six technical replicates each for seed collected from two plants. There was no statistical difference between WT and transgenic lines.

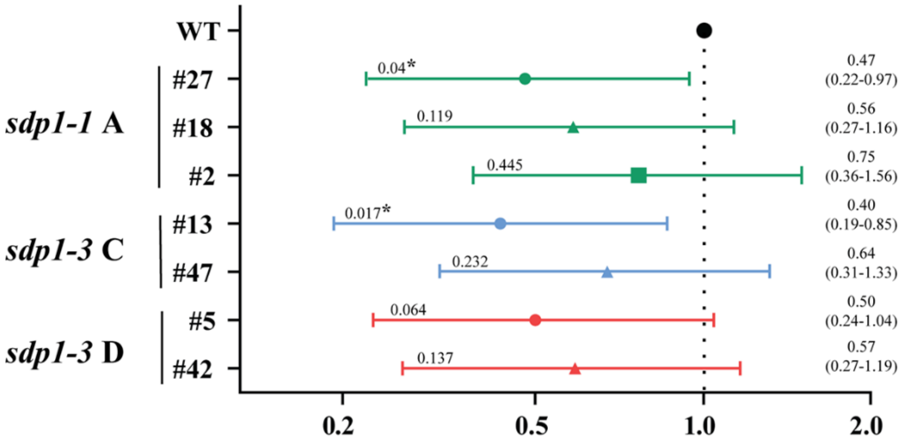

Figure S3. Cox proportional hazard model indicating hazard co-efficient of germination risk. Hazard ratios of estimated error, lower and upper value of estimated difference at 95% confidence interval and *p* values of germination risk of *GmSDP1*-suppressed lines as compared to the wild-type (WT).

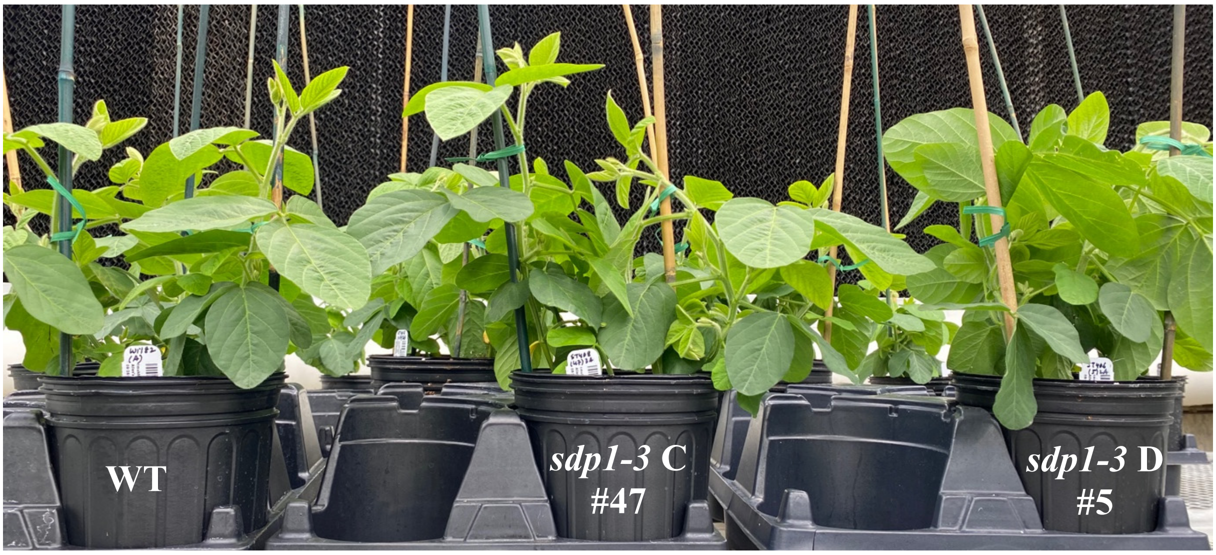

Figure S4. *GmSDP1*-suppressed lines develop similar to wild-type control plants. Representative four weeks old wild-type (WT) and *GmSDP1*-suppressed lines grown under greenhouse conditions.

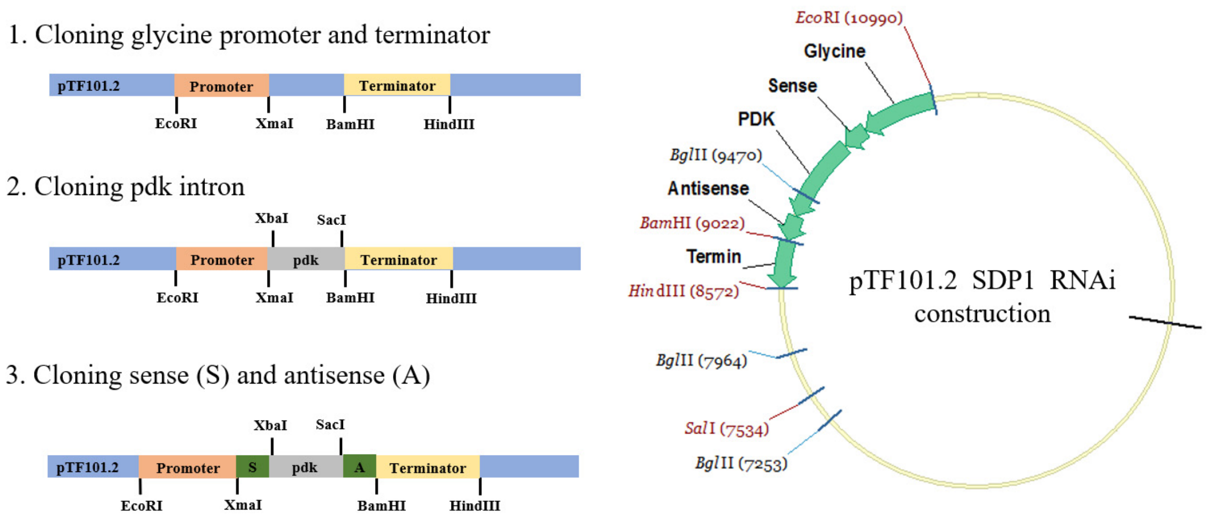

Figure S5. Scheme for the creation of the RNAi constructs used to target *GmSDP1* expression. (**A**) The promoter and terminator are introduced in two steps into the vector via EcoRI/XmaI and BamHI/HindIII respectively. (**B**) The PDK intron contained within the RNAi hairpin was amplified with specific primers including two restriction sites XmaI/XbaI and SacI/BamHI for each side and introduced in the vector. (**C**) The sense (S) and antisense (A) sequences were then cloned into the appropriate sites in the vector.

Gene	*sdp1-1 RNAi A*	*sdp1-1 RNAi B*	*sdp1-3 RNAi C*	*sdp1-3 RNAi D*
GmSDP1-1 **Glyma.02g190000**	100	100	90.0	90.0
GmSDP1-2 **Glyma.10g105200**	98.8	97.3	92.0	89.0
GmSDP1-3 **Glyma.19g132900**	89.6	90.6	100	100
GmSDP1-4 **Glyma.03g130900**	91.2	92.0	99.1	98.4

Table S1. Percent homology between each RNAi target sequence and *GmSDP1* paralogs.

		C16:0		C18:0		C18:1		C18:2		C18:3	
		**MEAN**	**±SD**	**MEAN**	**±SD**	**MEAN**	**±SD**	**MEAN**	**±SD**	**MEAN**	**±SD**
WT		13.7	0.41	4.2	0.23	19.6	1.16	54.8	1.35	7.7	0.26
sdp1-1 A	2	12.7	0.29	3.7	0.18	21.1	1.35	55.4	0.80	7.1	0.36
	12	13.2	0.33	4.2	0.16	19.3	0.93	55.8	0.63	7.5	0.42
	18	12.3	0.37	3.7	0.16	24.3	2.84	53.0	2.04	6.7	0.64
	27	12.4	0.32	3.8	0.14	23.3	2.22	53.7	1.77	6.9	0.43
	35	12.5	0.48	3.7	0.13	22.4	2.50	54.3	1.93	7.1	0.40
	52	13.1	0.41	4.0	0.23	22.2	1.46	54.0	1.48	6.7	0.26
sdp1-1 B	7	12.5	0.37	4.0	0.29	24.5	2.08	52.1	1.92	6.8	0.35
	23	12.7	0.33	3.7	0.13	20.4	2.43	55.7	2.10	7.4	0.72
	27	12.8	0.37	3.9	0.15	22.4	1.74	54.0	1.26	6.8	0.55
sdp1-3 C	12	12.7	0.46	3.8	0.20	20.6	1.47	54.9	0.94	8.0	0.50
	13	12.9	0.25	3.8	0.14	20.7	1.83	55.2	1.52	7.4	0.54
	15	12.9	0.32	3.9	0.20	20.9	1.25	55.1	0.74	7.2	0.50
	31	13.3	0.51	3.8	0.19	20.8	0.88	54.6	0.74	7.6	0.40
	47	13.4	0.26	4.0	0.17	20.8	1.19	54.3	0.83	7.4	0.52
	67	13.2	0.26	4.0	0.12	21.8	1.52	53.8	1.41	7.3	0.30
sdp1-3 D	5	12.4	0.44	3.8	0.37	23.9	3.12	53.1	2.83	6.8	0.57
	13	12.7	0.42	3.7	0.16	20.7	0.88	55.2	0.72	7.6	0.45
	16	12.5	0.74	3.8	0.31	24.1	1.70	52.6	1.92	7.0	0.45
	42	13.1	0.60	3.9	0.32	23.5	1.39	52.8	1.20	6.7	0.28

Table S2. Mean fatty acid content of T_3 seed harvested from transgenic lines targeting *GmSDP1*. Seeds from two plants were analyzed for each genotype. Shaded data is for lines shown in Figure 4B.

Name	Sequence 5' → 3'
GmSDP1-1For	TTG TCC AGG ACT TAG AAT TTC ATA AAG
GmSDP1-1Rev	TCG TCT CAG ACC ACC TAT TAA C
GmSDP1-2For	AAA TGG CAA CAG GAT GAT TGC C
GmSDP1-2Rev	ATT CCT ATT TGA AGG GGT CAA AAA GTC
GmSDP1-3For	TAG ATG CTG CTA GCT CAG CTT C
GmSDP1-3Rev	TGC TCT GAT CCA TAC CCG AAT G
GmSDP1-4For	TGA TAG CTC TGC ATC TGA ACA C
GmSDP1-4Rev	TGA TCC ATA CCC GAA TCT GTC C
ATP-For	TTT GCT GAA GCC TGT GGA GAT ATG
ATP-Rev	ACA ACC AAA TCA GTT TTC CCT CTG TG
SKIP16-For	AAC ATG GTC GCC GTT TAG AAC AC
SKIP16-Rev	CAT CTT GAA GAT CCA TCA ACT CAG GG
ELF1B-For	GCT TGA CAG TTG AGC CAT GC
ELF1B-Rev	ACA GAC TGA TCA GCC GCT TG

Table S3. Sequence of the oligonucleotides for RT-qPCR.

Name	Sequence 5' → 3'
pKMS3gly-F	GAATTCTACGTAAGTACGTACTCAA
pKMS3gly-R	AAGCTTAAGTCATGAAGAACCTGATAAGAC
pKMS3glyprom-F	GGATCCAGCCCTTTTTGTATGTGCTAC
pKMS3glyprom-R	CCCGGGGGTGATGACTGATGAGTGTTTAAG
Pdk-F	**CCCGGGTCTAGA**CCAATTGGTAAGGAAATAATTATTTTC
Pdk-R	**GGATCCGAGCTC**TTCGAACCCAATTTCCCAACTGTAATC
i_SDP1-1a senseF	CCCGGGGCATTTGGGAGGAC
i_SDP1-1a senseR	TCTAGAACCCTCTTCACAACTGTAAAAATCCC
i_SDP1-1a antisF	GAGCTCACCCTCTTCACAACTG
i_SDP1-1a antisR	GAGCTCGCATTTGGGAGGACTGCTT
i_SDP1-1b senseF	CCCGGGAGATGTCTTAACTACTTGAC
i_SDP1-1b senseR	TCTAGATGCATCATAGGTAAATCATTCTCCAAGC
i_SDP1-1b antisF	GAGCTCTGCATCATAGGTAAATCATTCTCC
i_SDP1-1b antisR	GGATCCAGATGTCTTAACTACTTGACTTCACC
i_SDP1-3c senseF	CCCGGGATTATGTGTGCTGTTGTTG
i_SDP1-3c senseR	TCTAGACCTGTCATGTCATAAGCTTCTTG
i_SDP1-3c antisF	GGATCCCCTGTCATGTCATAAGCTTC
i_SDP1-3c antisR	GGATCCATTATGTGTGCTGTTGTTGCC
i_SDP1-3d senseF	CCCGGGCGCCGTTGGAG
i_SDP1-3d senseR	TCTAGAGCAAGTCCACCTAATGGAAAACC
i_SDP1-3d antisF	GAGCTCGCAAGTCCACCTAATGGAAAACC
i_SDP1-3d antisR	GGATCCCGCCGTTGGAGGGATG

Table S4. Sequence of the oligonucleotides for RNAi construction.
